# Isolation, identification, and phylogenetic analysis of subgroup III strain of bovine respiratory syncytial virus contributed to outbreak of acute respiratory disease among cattle in Northeast China

**DOI:** 10.1080/21505594.2021.1872178

**Published:** 2021-01-20

**Authors:** Shuo Jia, Xin Yao, Yaqi Yang, Chao Niu, Yi Zhao, Xiaomei Zhang, Ronghui Pan, Xiaoxia Jiang, Sun Xiaobo, Xinyuan Qiao, Xueting Guan, Yigang Xu

**Affiliations:** a Heilongjiang Province Key Laboratory for Animal Disease Control and Pharmaceutical Development, Northeast Agricultural University, Harbin, China; b Veterinary Laboratory, Jilin Province Centre for Animal Disease Control and Prevention, Changchun, China; c Northeastern Science Inspection Station, China Ministry of Agriculture Key Laboratory of Animal Pathogen Biology, Harbin, China

**Keywords:** Subgroup III strain of bovine respiratory syncytial virus, isolation and identification, phylogenetic analysis, Northeast China

## Abstract

Bovine respiratory syncytial virus (BRSV) is a clinically important causative agent of acute respiratory diseases in postweaning calves and feedlot cattle and causes numerous economic losses to the cattle industry. In June 2018, an outbreak of an acute respiratory disease occurred among 4- to 10-month-old calves on three intensive beef cattle farms in Heilongjiang Province, Northeast China, with a 27.42% morbidity rate (329/1200) and a > 25% mortality rate (85/329). Using next-generation sequencing, we comprehensively analyzed microbial diversity in the lung samples of the diseased cattle and found that the causative agent of this epidemic outbreak is mainly a bovine orthopneumovirus named BRSV strain DQ. We then isolated and confirmed the virus by RT-PCR and an indirect immunofluorescence assay. Phylogenetic analysis of genes *G, F, N, NS1, NS2*, and *SH* of BRSV strain DQ showed that this strain shares the highest genetic similarity with strains USII/S1, 15489, V41, and NY487834 belonging to subgroup III of BRSV. This is the first report of subgroup III strain of BRSV presence in China. Heilongjiang Province is a major cattle-breeding province in China; therefore, it is necessary to test for BRSV in the cattle trade and to conduct region-extended epidemiological surveillance for BRSV in China.

## Introduction

Bovine respiratory syncytial virus (BRSV) is the major causative agent of acute respiratory diseases in postweaning calves and feedlot cattle during the first year of life and is also one of the main causes of the bovine respiratory disease complex in dairy and beef calves [1,2]. BRSV can be transmitted directly from infected, symptomatic animals to others or by aerosols, thereby causing serious respiratory system disease with clinical symptoms of fever, nasal discharge, cough, and even death [3,4]. The morbidity rate of BRSV infection in calves is relatively high and can reach 60%, while the fatality rate is approximately 20%, causing substantial economic losses to the cattle industry worldwide [4]. Sometimes, coinfections of calves by BRSV and other pathogens can cause severe respiratory symptoms. In 2017, a beef cattle feedlot in Southeastern Brazil suffered coinfection by BRSV and the bacterium, *Histophilus somni* [5]. In 2018, coinfection by BRSV and bovine parainfluenza virus 3 was identified in Turkey [2]. Generally, based on the sequence variability of the gene encoding the G protein of BRSV, BRSV is classified into seven genetic subgroups (I to VII) [3], and different BRSV subgroups are prevalent in different countries or regions [3,6]. BRSV infections are considered a major cause of bovine respiratory diseases in the United States and Europe, and although most infections are unapparent, the high prevalence of seropositive cattle indicates that the rate of BRSV infection is still high [6].

Similar to the human respiratory syncytial virus, BRSV belongs to the genus *Orthopneumovirus* of the *Pneumoviridae* family and harbors an approximately 15 k bp-long single-stranded negative-sense RNA genome [7,8], which encodes 11 proteins including three surface glycoproteins (glycoprotein G, small hydrophobic protein SH, and fusion protein F), nucleoprotein (N), viral RNA-dependent polymerase protein (L), phosphoprotein (P), matrix protein (M), transcriptional anti-termination factor M2-1, RNA regulatory protein M2-2, and two nonstructural proteins, namely, NS1 and NS2 [9,10]. Since the 1990s, molecular-genetic characterization studies have revealed that the emergence of new variants manifests a geographic correlation [6]. Among them, subgroup I consists of European strains (the UK and Switzerland); subgroup II contains strains isolated in Belgium, France, Denmark, Sweden, Japan, and the Netherlands; subgroup III includes strains isolated mainly in the USA; subgroup IV of BRSV comprises European and USA strains; subgroups V and VI contain only French and Belgian isolates [2,6]; and subgroup VII of BRSV includes only Italian isolates [3]. BRSV was first reported in Geneva in 1970 and has currently spread worldwide, including the United States, Japan, Belgium, Norway, Canada, Italy, Denmark, France, Brazil, and China, as a result of live-animal or animal-product exports [2,11,12]. According to a phylogenetic tree, researchers have reported that BRSV strains circulating in the cattle population of the Czech Republic are more closely related to the Danish strains from the 1995 outbreak, thus suggesting that animal trade may be a route of BRSV transboundary transmission [13].

Heilongjiang Province is a major cattle-breeding province in China. Before 2019, the general cattle population in Heilongjiang Province was over six million including dairy cattle and beef cattle. For most intensive cattle farms in the Heilongjiang Province, breeding cattle, frozen semen, and/or embryos are mainly imported from Australia, New Zealand, the United States (frozen semen), Canada, and Uruguay. In June 2018, there was an outbreak of an acute respiratory disease among postweaning calves and feedlot beef cattle on several beef-cattle farms located in the Heilongjiang Province, Northeast China, with clinically significant signs and symptoms, such as anorexia, high body temperature, nasal secretions (runny nose), and salivation accompanied with a cough. More than 300 cattle were affected by the disease with a mortality rate of more than 25% (85/329). The use of antibiotics such as fluoroquinolones, streptomycin, and gentamicin had no therapeutic effect on the diseased cattle. Moreover, possible bacterial pathogens such as *Pasteurella multocida, Staphylococcus aureus*, and *Streptococcus pneumoniae* were not detectable by culture techniques. It is well known that molecular methods are becoming the gold standard for accurate identification and genetic analysis of pathogens [6,10]. Among these methods, next-generation sequencing technology with its rapidity and high-throughput characteristics is becoming an important technique for identifying known or unknown pathogens in clinical samples [14–17]. Therefore, in this work, next-generation sequencing was employed to identify the causative agent contributed to the outbreak of acute respiratory disease among cattle, followed by isolation and biological characteristic analysis.

## Materials and methods

### Disease outbreak and sample collection

**Figure 1.**
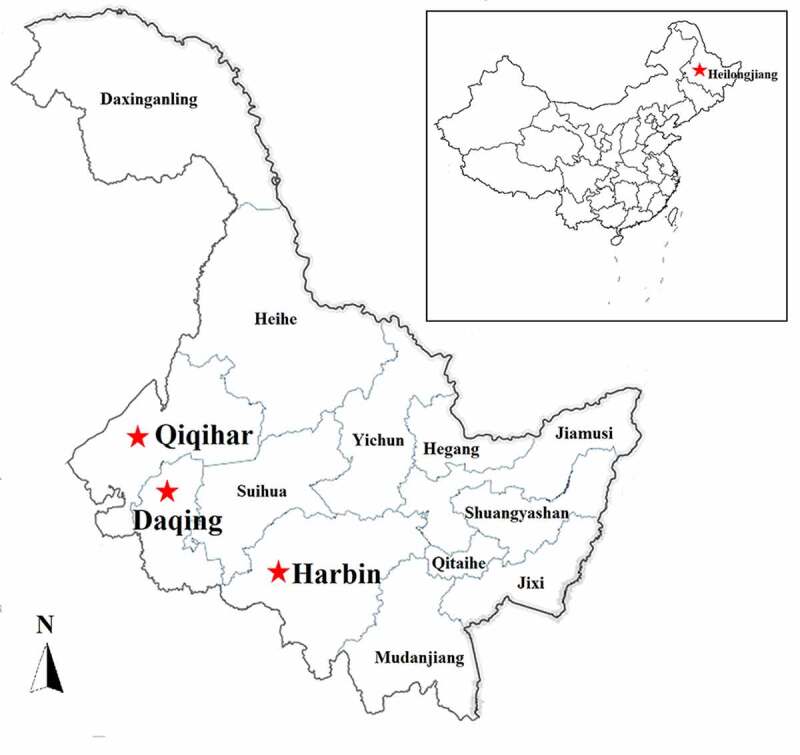
Outbreak of an acute respiratory disease among postweaning calves and feedlot beef cattle on cattle farms located in Heilongjiang Province, Northeast China, in June 2018

**Figure 2.**
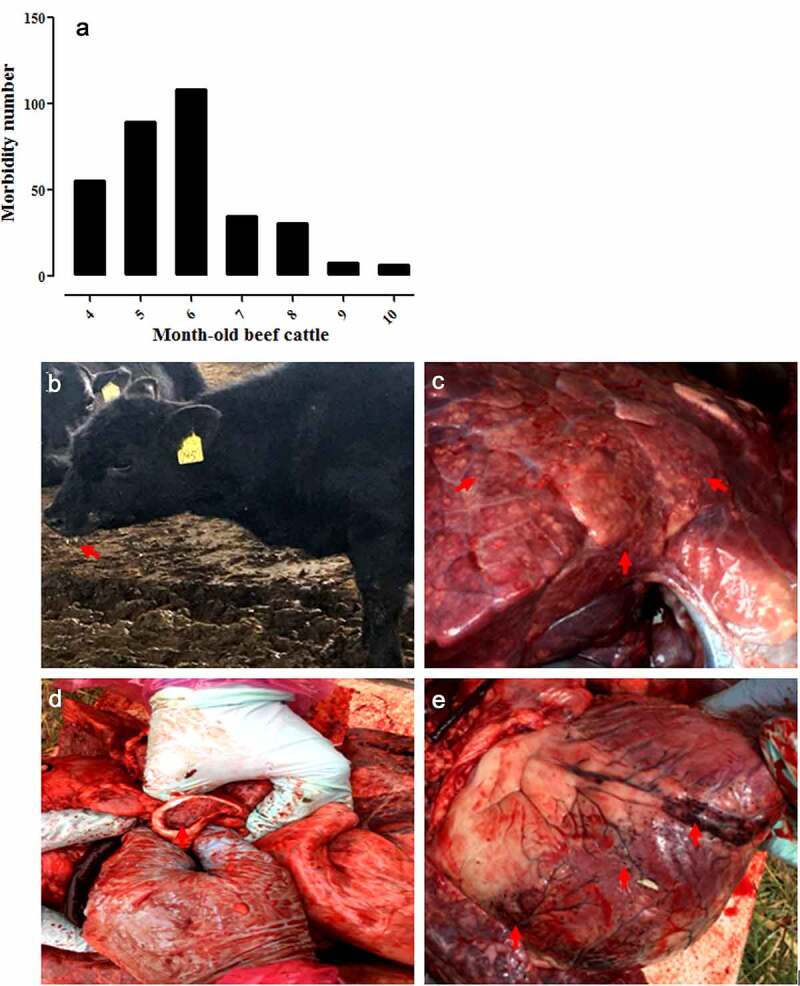
Number of diseased animals among 4–10-month-old feedlot beef cattle in this epidemic (a), and clinically significant signs and symptoms observed in the diseased cattle, including salivation (b), vacuole-like emphysematous lesions in the lungs (c), trachea filled with secretions (d), and punctate hemorrhages of the heart (e), which were marked by the red arrows

In June 2018, an acute-respiratory-disease outbreak occurred among 4- to 10-month-old postweaning calves and feedlot beef cattle on three beef cattle farms located in Daqing, Harbin, and Qiqihar of the Heilongjiang Province (Figure 1). On these farms, there were no vaccination records for the last 3 years regarding vaccines against BRSV, bovine viral diarrhea virus, and infectious bovine rhinotracheitis virus. During this epidemic, 329 beef cattle (4–10 months old) were affected by the disease (Figure 2a) and the total morbidity rate was 27.42% (329/approximately 1200) with a mortality rate of >25% (85/329). Clinically significant signs and symptoms of the diseased cattle included a fever, anorexia, salivation (Figure 2b), cough, and death. The main histopathological changes were vacuole-like lesions in the lungs (Figure 2c), trachea filled with secretions (Figure 2d), and punctate hemorrhages in the heart (Figure 2e). Under the conditions ensuring biological safety, lung tissues of the diseased cattle were collected for next-generation sequencing analysis. The collection of experimental samples was conducted in accordance with the international (OIE Terrestrial Animal Health Code) and national guidelines (CNAS-CL06:2018) for the care and use of laboratory animals. The project (No. 2018NEAU06423) was approved by the Committee on the Ethics of Animal Experiments of the Northeast Agricultural University of China (29 June 2018).

### Next-generation sequencing

Eighteen lung tissue samples were collected randomly from the diseased cattle on each farm (six lung samples per farm) and placed on dry ice and sent directly to BGI Genomics Co. Ltd. (China) for sequencing and data analysis. Following total RNA extraction and quality inspection, the library preparation was carried out using the total RNA (2 μg) without rRNA, and the sample sequencing was performed using Illumina HiSeq4000 sequencing platform (Illumina, San Diego, CA, USA) followed by data analysis. To ensure the results more reliable, the raw data were cleaned by removing the adaptor sequence, the reads containing more than 20% bases with a quality value <15, and the reads whose total number of N-containing bases reach 5%. Then, the remaining reads were blasted with the host (bovine) database to remove the reads that may be host contamination (the reads with a comparison consistency over 90% are regarded as host contamination). The filtered reads were assembled de novo using the Trinity software, and then all the sequences were integrated. Next, following redundancy elimination (the sequence identity threshold of 0.95) by CD-HIT-EST software, the unigenes were obtained. Subsequently, using the unigene obtained as a reference, mapping the sequencing reads to the unigene by hisat2 software, and comparing the blast results by htseq-count software, the abundance (read count) and expression level of each unigene was obtained. After that, assembled unigenes were annotated using BLAST alignment (e-value<1e-5), followed by species annotation using LCA algorithm of MEGAN software.

### RT-PCR, virus isolation and IFA

According to the results of next-generation sequencing, BRSV was the main causative agent contributed to the outbreak of acute respiratory disease among cattle in the Heilongjiang Province. Next, diagnostic reverse-transcription polymerase chain reaction (RT-PCR) testing for the presence of BRSV in these lung samples was performed. The total RNA of lung samples was extracted using the TRIzol Total RNA Isolation Kit (Invitrogen, Grand Island, NY, USA) *[18]*, and then first-strand cDNA was synthesized using QuantiTect Reverse Transcription Kit (Qiagen, Hilden, Germany). Briefly, template RNA (up to 1 μg), gDNA wipeout Buffer 2 μL, and RNase-free water added to 14 μL were mixed in a centrifuge tube, incubated at 42°C for 2 min, and placed on ice immediately. After that, Quantiscript Reverse Transcriptase 1 μL, Quantiscript RT Buffer 4 μL, and RT Primer Mix 1 μL were added into the tube and incubated at 42°C for 15 min, followed by incubation at 95°C for 3 min to inactivate Quantiscript Reverse Transcriptase. After that, using the cDNA as a template, BRSV was detected by PCR with primers targeting the *N* gene of BRSV (Table 1). The thermal cycling conditions were as follows: 95°C for 5 min; 35 cycles of 94°C for 30 s, 55°C for 30 s, and 72°C for 35 s; and final extension at 72°C for 10 min.

**Table 1.** Primers used in this study

Gene	Primers (5ʹ→3ʹ)	Product size (bp)	Tm (°C)
G	F: ATGTCCAACCATACCCATCATAT R: TTAGATCTGTGTAGTTGATTGATTTATGTG	774	58
F	F: ATGGCGGCAATAGCCATAAGGATG R: TCATTTACTAAAAGAAAGATTGTTGATACC	1725	52.5
N	F: ATGAGCACCCCAAGTCCCGAGAC R: TCACAATTCCACATCATTATCTTTGG	1176	55
NS1	F: ATGGGCAGTGAAACATTGAGTGT R: CTAATTCAGACCAAAAAGAATAG	411	56
NS2	F: ATGAGCACCCCAAGTCCCGAGAC R: TCCCTATGGATTTAAATCATACTTG	378	52
SH	F: ATGAACAGTACATCTACCATAATAAAG R: TTAACTTTGATGCATGTTGC	291	53

Next, the lung tissue samples (0.5 g per sample) were fully ground in liquid nitrogen, followed by the addition of Dulbecco’s Modified Eagle’s Medium (DMEM; Gibco, Grand Island, NY, USA), mixing, and centrifugation at 12,000 × *g* for 10 min. The supernatant was inoculated onto a confluent monolayer of Madin-Darby bovine kidney (MDBK) (NBL-1) cell that was purchased from the American Type Culture Collection (ATCC) and kept in our laboratory) and incubated at 37°C in a 5% CO_2_ incubator for 1 h. After discarding the supernatant, MDBK cells were gently washed twice with sterile phosphate-buffered saline (PBS) at pH 7.2, followed by the addition of maintenance DMEM without fetal bovine serum. The cells were continually incubated at 37°C in a 5% CO_2_ incubator until the cytopathic effect exceeded 80%. MDBK cells mock-infected with DMEM served as a control. The isolated virus was identified using the RT-PCR method described above.

Then, an indirect immunofluorescence assay (IFA) was carried out to identify BRSV propagated in MDBK cells. Briefly, after inoculation for 24 h, virus-infected MDBK cells and mock-infected MDBK cells were fixed with 4% paraformaldehyde at room temperature (RT) (20–25°C) for 15 min, permeabilized with 0.2% Triton X-100 at RT for 10 min, and blocked with 0.3% bovine serum albumin at 37°C for 30 min. Next, a mouse monoclonal antibody against the G protein of BRSV (prepared in our laboratory) as the primary antibody and a fluorescein isothiocyanate (FITC)-conjugated goat anti-mouse IgG antibody (1:300; Abcam, Cambridge, MA, USA) as the secondary antibody was incubated with the MDBK cells, in that order. After two washes with sterile PBS, the cells were examined under a fluorescence microscope (Bio-Rad, Hercules, CA, USA) [19].

### Phylogenetic analysis

To verify the results of next-generation sequencing and to explore the evolutionary relationships between the BRSV strain isolated in this work and other BRSV strains published in the GenBank, genes *G, F, N, NS1, NS2*, and *SH* of the BRSV strain isolated in this work were amplified using RT-PCR with the primers (Table 1) designed according to the result of the next-generation sequencing. The full-length gene sequence was subcloned into plasmid pMD-19 T [20] followed by sequencing analysis. The genome sequence of the BRSV strain and the sequences of its genes encoding proteins G, F, N, NS1, NS2, and SH were deposited in GenBank under accession numbers MT861050, MN316655, MN316656, MN316653, MN316651, MN316652, and MN316654, respectively. Then, for the genes, *G, F, N, NS1, NS2*, and *SH*, cognate sequences from reference BRSV strains representing different subgroups (listed in Table 2) were retrieved from the GenBank database by means of the BLAST engine, and phylogenetic analysis was performed on the genes *G, F, N, NS1, NS2*, and *SH* by the neighbor-joining method (1000 replicates) using MEGA 7 software [21]. After that, alignments of amino acid sequences based on the *G* gene of the BRSV strain identified in this study and other reference BRSV strains from different subgroups were conducted using the DNAMAN software [21].

**Table 2.** The main reference BRSV strains used in this study

Subgroup	Strain	Country	Year	Gene	Accession No.
I	127	UK	1973	G	Y08716
I	4642	UK	1976	F,G	Y08718
I	A51908	USA	1975	whole	AF295544
II	HU03	Czech Republic	2003	G	AY507914
II	FV160	Belgium	1969	F,G	AF188578
II	22,069	Belgium	1969	F	AF188575
II	88Lu195	Denmark	1988	NS1, NS2, N, SH	AF054664
II	9,314,893	Denmark	1993	NS1, NS2, N, G, SH	AF054668
II	9,304,899	Denmark	1993	NS1, NS2, N, G	U92101
II	9,510,070	Denmark	1995	G, SH	AF248585
II	9,402,020	Denmark	1994	NS1, NS2, N, G	U92103
II	9,402,022	Denmark	1994	G, SH	U92104
II	9,514,923	Denmark	1995	G, SH	AF248588
II	W2	Belgium	1969	F, N	AF188572
II	LELYSTAD	The Netherlands	1974	F, N, G	AF188552
II	HPIG-SLU-620-Lovsta	Sweden	2016	whole	MG947594
II	9,510,236	Denmark	1995	G, SH	U92107
II	9,513,317	Denmark	1995	G, SH	AF248586
II	9,610,783	Denmark	1996	G, SH	AF248589
II	9,616,957	Denmark	1996	G, SH	AF248590
II	9,810,829	Denmark	1998	G, SH	AF248596
II	9,911,060–1	Denmark	1999	SH	AF248581
II	9,910,130	Denmark	1999	G, SH	AF248597
II	NMK7	Japan	1968	G	U24713
III	A2Gelfi	France	1995	F, G, N	AH008996
III	FS-1	USA	1975	G	AF188581
III	391–2	USA	1985	F, G, N, M	S40504
III	85–1330	USA	1985	G	U24716
III	V27	USA	1986	G	AY910758
III	V30	USA	1987	G	AY910762
III	V41	USA	1988	G	AY910756
III	USII/S1	USA	2015	whole	KU159366
III	BAYOVAC	USA	–	F, N	AF188571
III	NY487834	USA	–	G	AY910755
III	15,489	USA	-	G	AY910757
III	IT135	Italy	2013	N, G	KY753450
III	IT22152	Italy	2015	N, G	KY753448
III	IT6167A	Italy	2015	N, G	KY753452
III	W26	–	–	G	AY910760
III	375	–	–	F, G	AF188579
IV	90,504	France	1990	F, G, N	AF188580
IV	Dorset	UK	1971	G	U24715
IV	SNOOK	UK	1976	G	Y08719
IV	ATCC51908	USA	1975	whole	NC_038272
IV	5761	Belgium	1990	F, G, N	AF188583
IV	WBH	The Netherlands	1986	G	Y08717
IV	V347	The Netherlands	1990	F, G	AF188584
IV	9,617,058	Denmark	1996	G, SH	AF248591
IV	9,710,455–1	Denmark	1997	G, SH	AF248592
IV	9,710,985	Denmark	1997	G, SH	AF248594
IV	9,910,244	Denmark	1999	G, SH	AF248601
IV	ATue51908	Germany	1998	whole	AF092942
V	W6	France	1993	F, G, N	AF188595
V	394	France	1995	F, G	AF188596
V	A1	France	1995	F, G	AF188555
V	BIOCH	France	1995	N	AF188540
V	B2	France	1996	F	AF188557
V	C2	France	1996	F, G	AF188558
V	B1	France	1996	F, G, N	AF188589
V	D1	France	1996	N	AF188542
V	C1	France	1996	G, N	AF188590
V	F2	France	1997	F	AF188564
V	L1	France	1997	F	AF188563
V	I1	France	1997	G, N	AF188545
V	G2	France	1997	F, G, N	AF188544
V	J1	France	1997	F, G, N	AF188592
V	88P	France	1998	F, G, N	AF188604
V	58P	France	1998	G, N	AF188603
V	P8	Belgium	1998	F, G	AF188567
V	P4	Belgium	1998	N	AF188532
VI	K1	France	1997	F, G, N	AF188585
VI	K2	France	1997	G	AF188586
VII	IT24374	Italy	2012	N, G	KY753465
VII	SM56243	Italy	2012	N, G	KY753461
VII	IT1299	Italy	2013	G	KY753463
	88CVA70	–	–	NS1, NS2, N	AF054665
	KIM24	–	–	N	AF188551
	BRU15937	–	–	NS1	U15937
	BRU15938	–	–	NS2	U15938

## Results

### Identification of the causative agent of the acute-respiratory-disease outbreak among the cattle by the next-generation sequencing technology

**Figure 3.**
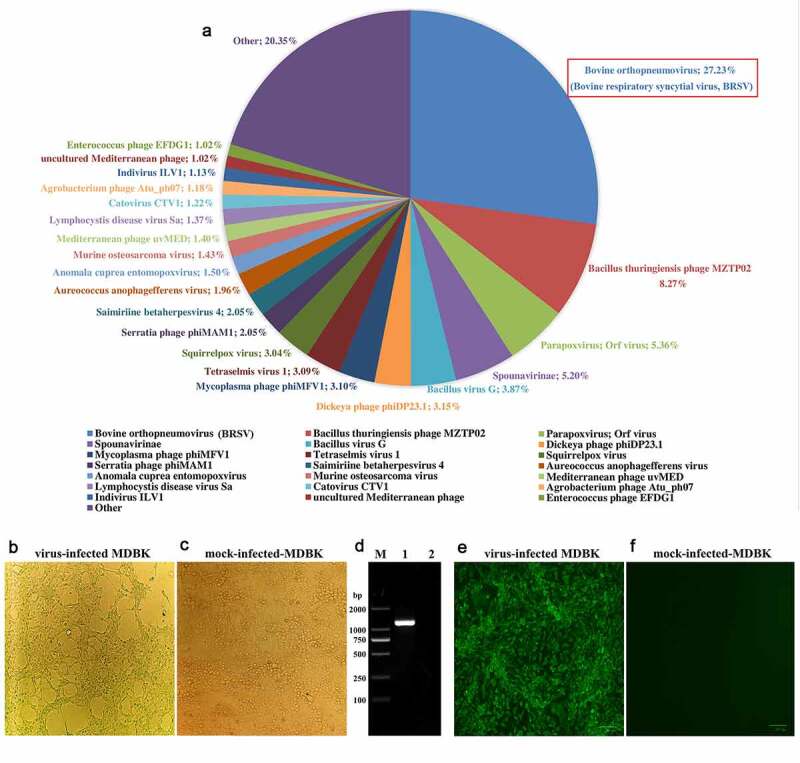
Viral pathogens in lung tissue of diseased cattle were detected by next-generation sequencing (a). BRSV strain DQ was isolated using MDBK cells and an obvious cytopathic effect was observed in these cells (b) but not in mock-infected MDBK cells (c). BRSV strain DQ propagated in MDBK cells was identified by RT-PCR (d) (lane M: DNA molecular weight markers DL 2000, lane 1: the BRSV strain DQ propagated in MDBK cells, and lane 2: mock-infected control) and indirect immunofluorescence assay with mouse anti-BRSV-G protein monoclonal antibody at 24 h post-infection (e), using mock-infected MDBK cells as a negative control (f)

**Figure 4.**
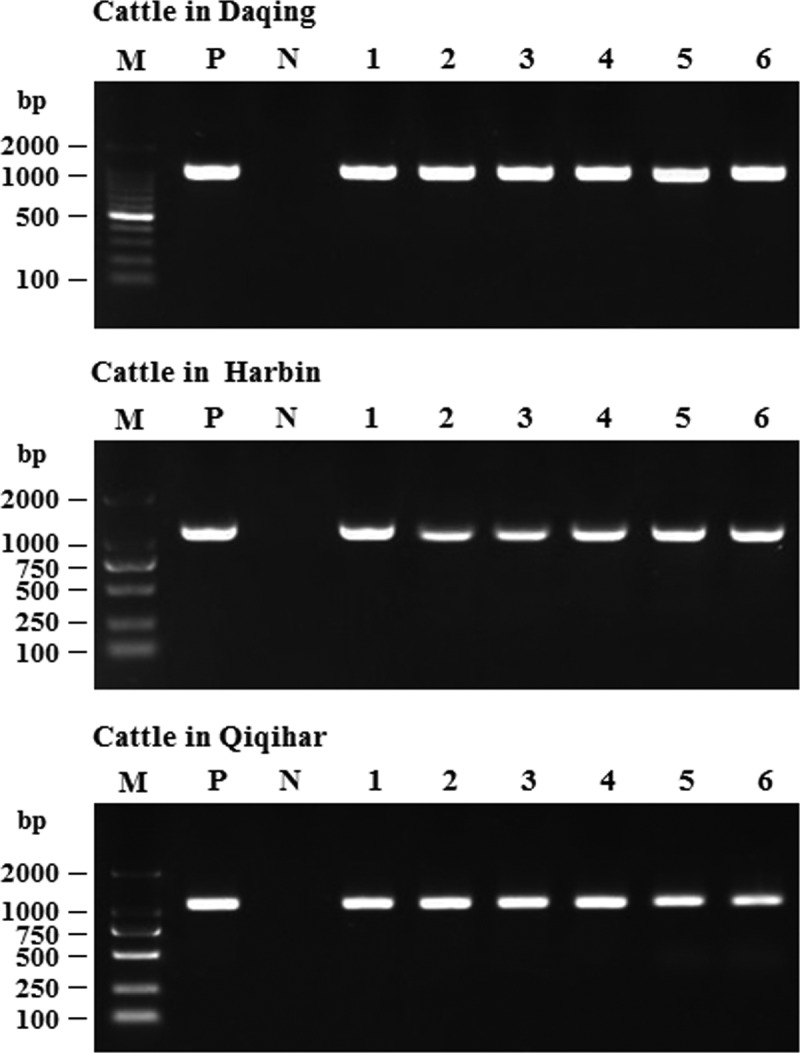
Diagnostic RT-PCR targeting N gene of BRSV was performed on lung samples (from the diseased cattle collected on each farm) that were BRSV positive according to next-generation sequencing. M: DNA molecular weight markers, P: positive control, and N: negative control

As already mentioned, in June 2018, the acute-respiratory-disease outbreak occurred among postweaning calves and feedlot beef cattle in Heilongjiang Province. Because antibiotics had no therapeutic effect on the diseased cattle, we speculated that the disease was caused by viral pathogens. In recent years, the next-generation sequencing technology has been widely used to detect unknown pathogens in clinical samples [22,23] and is a more rapid and efficient assay than PCR-based molecular diagnostic methods [24]. Therefore, we employed the next-generation sequencing to identify potential causative agents of the acute-respiratory-disease outbreak. Our results showed that a bovine orthopneumovirus (BRSV) accounted for approximately 25–30% of the total number of viral pathogens in all lung-tissue samples collected from diseased cattle, which was the main causative agent contributed to the disease outbreak. Results of next-generation sequencing obtained from a representative animal are shown in Figure 3a. After an integrated genome assembly of BRSV detected in each lung sample, our data showed that the complete genome sequence is 15,151 bp in length encoding 11 predicted proteins. After the whole genomic sequence alignment of the BRSV strains from the lung samples of these diseased cattle, we found that their homology was almost 100%. Therefore, we named the causative agent as BRSV strain DQ. In addition, a specific diagnostic RT-PCR test for BRSV in these lung samples was performed (Figure 4) with the primers designed according to the next-generation sequencing results. All lung samples were confirmed to be BRSV positive. Although next-generation sequencing may not be commonly carried out by all diagnostic laboratories, it actually plays an important part in rapid high-throughput identification of pathogens in clinical samples.

### Isolation of BRSV strain DQ

BRSV strain DQ was isolated from the lung tissue samples of diseased cattle using MDBK cells. Cytopathic effects were clearly detectable in the virus-infected MDBK cells (Figure 3b), but not in mock-infected MDBK cells (Figure 3c), after 36 h of incubation. Next, the virus was detected using RT-PCR targeting the *N* gene of BRSV and the result showed that the virus propagated in MDBK cells was positive for this gene (Figure 3d). After gene sequencing and BLAST analysis, our data indicated that the homology of the *N* gene between the BRSV DQ strain and known reference BRSV strains (accession numbers AF054668, AF092942, AF054665, U92103, MG947594, NC038272, and AF054664) published in GenBank was as high as 99.27%. In addition, the isolated virus was identified using an immunofluorescence assay with a mouse monoclonal antibody against the G protein of BRSV, and the finding revealed a remarkable green fluorescence in the virus-infected MDBK cells (Figure 3e), but not in the mock-infected MDBK cells (figure 3f), indicating that the virus could be specifically recognized by the anti-BRSV-G antibody.

### Phylogenetic analysis

**Figure 5.**
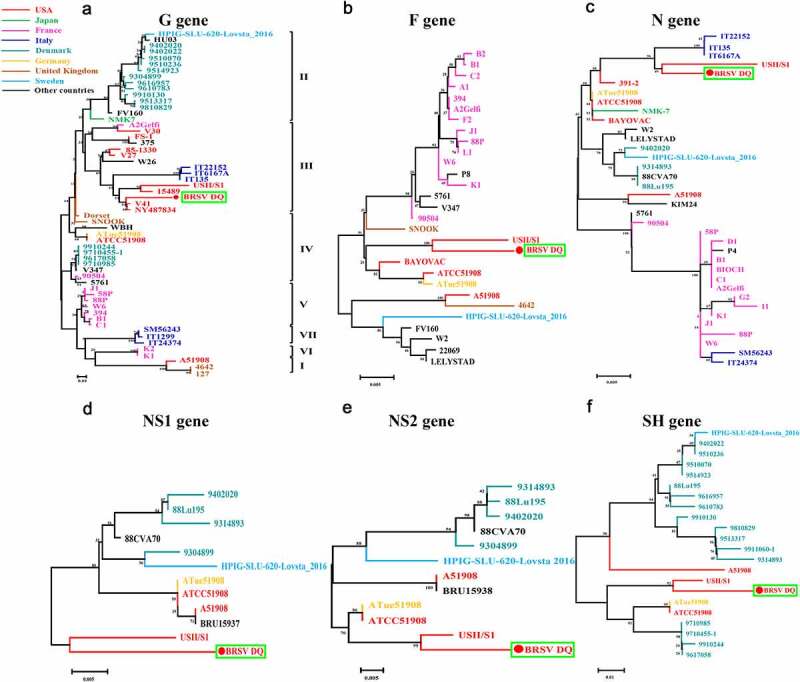
Phylogenetic analysis of genes *G, F, N, NS1, NS2*, and *SH* between BRSV strain DQ and other BRSVs found in GenBank. The sequences of *G, F, N, NS1, NS2*, and *SH* genes from reference BRSV strains representing different subgroups were retrieved from the GenBank database by means of the BLAST engine, and phylogenetic analysis was performed on the genes *G, F, N, NS1, NS2*, and *SH* by the neighbor-joining method (1000 replicates) using MEGA 7 software

**Figure 6.**
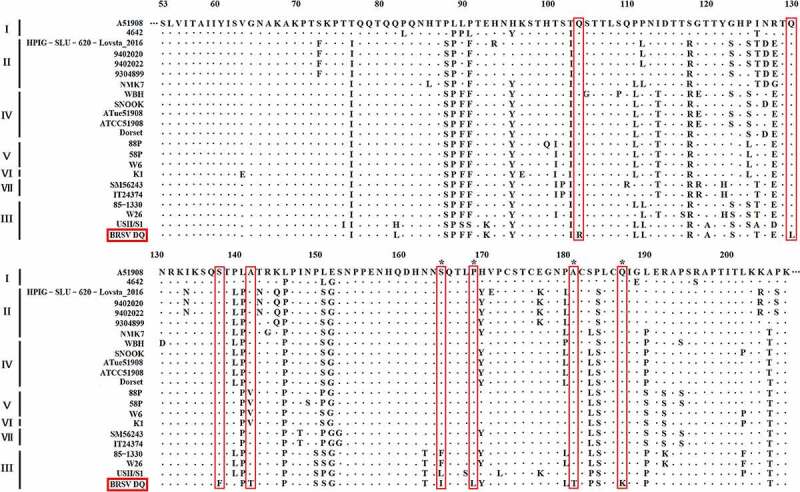
Multiple sequence alignment of amino acid sequences of the G protein between BRSV strain DQ and other main reference BRSVs (those published in GenBank) was conducted using the DNAMAN software

Following the amplification of the genes *G, F, N, NS1, NS2*, and *SH* of the BRSV strain DQ using RT-PCR and sequencing, phylogenetic analysis of these genes was performed using MEGA 7 software to explore the evolutionary relationships between the BRSV strain DQ isolated in this study and other BRSVs found in GenBank. As presented in Figure 5, the phylogenetic analysis showed that the *G* gene of BRSV strain DQ has the highest sequence homology to the USA strains USII/S1, 15489, V41, and NY487834. Moreover, the *F, N, NS1, NS2*, and *SH* genes show the highest sequence homology to the USA strain USII/S1, indicating that BRSV strain DQ has the closest genetic relationship with the USA strain USII/S1. In the United States, BRSV subgroup III strains have given rise to several bovine-respiratory-disease outbreaks [8,25,26]. Notably, according to the evolutionary relationships, BRSV strain DQ belongs to BRSV subgroup III, just as USA strains USII/S1, 15489, V41, and NY487834 do. This is the first report about the presence of BRSV subgroup III strain in China. Moreover, the amino acid sequence similarities of the G protein between BRSV strain DQ and other BRSVs identified as subgroup III strains ranged from 83.27–90.27% (Figure 6). Studies have revealed that amino acid residues 158–189 represent a central conserved region of BRSV protein G, and residues 174–187 of the central conserved region are immunodominant [9,27]. However, substitutions of residues 165 (Ser-Ile), 169 (Pro-Leu), 181 (Ala-Thr), and 187 (Gln-Lys) were found in this study. Although the antigenic reactivity of BRSV strain DQ was not analyzed in this study, we presume that these changes in antigenic epitopes may influence antibody reactivity and binding. Additional studies on antigenic properties of BRSV strain DQ should be further conducted to verify our hypotheses.

## Discussion

In China, a BRSV strain HJ was first isolated from diseased cattle in 2007 and was found to be closely related to the Japanese strain NMK-7 belonging to subgroup II [28]. In the present study, we isolated and identified a subgroup III strain of BRSV: strain DQ. To the best of our knowledge, this is the first report on the presence of subgroup III strain of BRSV in China. In recent years, there were many reports of BRSV infection in cattle worldwide, e.g., in the United States [26], Brazil [5], Italy [3], Turkey [2], Sweden [29], Poland [4], Canada [30], and Croatia [10], and this infection caused substantial economic losses to the cattle industry. In these reports, subgroup III BRSV strains mainly contributed to the outbreaks of bovine respiratory disease in feedlot cattle. In the United States, several bovine-respiratory-disease outbreaks were mainly caused by the BRSV subgroup III strains [8,25], such as the USII/S1 strain, which caused the infection in 2015 [26]. In Europe, BRSV infection was highly prevalent among cattle herds [29,31,32] and the main causative agents included BRSV subgroup III strains [3,33]. In this series of reports, in 2018, coinfection by BRSV subgroup III and bovine parainfluenza virus 3 was first identified in Turkey [2]. Moreover, Southeastern Brazil first reported coinfection by a BRSV subgroup III strain and *H. somni* in feedlot cattle in 2017 [5].

“What now” to control BRSV in China is a concerned issue. Many infectious diseases may spread among cities, countries, and continents as a result of live-animal or animal-product transportation. In China, the demand for beef in the domestic market is increasing annually and leading to an increase in the amounts of imported breeding cattle, frozen semen, and embryos coming from abroad, e.g., from Australia, New Zealand, the United States, Brazil, and Uruguay. Strictly implementing quarantine policy for imported live cattle and detection for genetic materials (frozen semen, embryos) plays an important role in preventing cross-border transmissions of infectious diseases, such as bluetongue disease, bovine spongiform encephalopathy, Akabane disease, bovine nodular skin disease, bovine leucosis, and bovine viral diarrhea. Nevertheless, BRSV is not on the quarantine list for imported cattle in China. Maybe, subgroup III strain DQ of BRSV was spread into Heilongjiang Province by animal trade. Further epidemiological survey confirmed that the genetic materials, in particular frozen semen, were important for potential transmission of the virus, thereby highlighting the necessity of detecting BRSV in imported cattle and genetic materials. Moreover, region-extended epidemiological surveillance of BRSV infection in China is recommended.

After the outbreak of the epidemic caused by BRSV strain DQ, we have taken positive measures to restrict the spread of this new viral strain, including daily elimination of feces, thorough disinfection (environments, appliances, and work clothes), elimination of diseased cattle, isolation of suspected cattle, and injection of an antiviral drug such as interferon combined with traditional Chinese medicine. By eliminating the source of infection and blocking the transmission route, we have effectively controlled the spread of the epidemic and further reduced economic losses to farmers. Currently, our research is underway to develop a vaccine against BRSV strain DQ infection.

In conclusion, an acute respiratory disease outbreak was caused by BRSV subgroup III strain DQ in calves from Heilongjiang Province, Northeast China, in 2018. This is the first report of BRSV subgroup III strain presence in China. Animal trade including live animal, animal product, and genetic materials was the main route for potential transmission of the diseases. Therefore, it is necessary to strengthen the detection of BRSV and other pathogens in animal trade and to perform region-extended epidemiological surveillance of BRSV infection in China.
